# Single‐cell RNA sequencing of pediatric renal tissues revealed the potential relationship between immunoglobulin A nephropathy and immunoglobulin A vasculitis with nephritis

**DOI:** 10.1002/ctm2.1651

**Published:** 2024-04-09

**Authors:** Qing Ye, Hanyan Meng, Fang Ye, Haidong Fu, Jingjing Wang, Fei Liu, Huijun Shen, Yu Bao, Chunyue Feng, Xiaojing Zhang, Guoping Huang, Yi Xie, Xiujuan Zhu, Manli Zhao, Guoji Guo, Jianhua Mao

**Affiliations:** ^1^ Department of Laboratory Medicine Children's Hospital, Zhejiang University School of Medicine, National Clinical Research Center for Child Health, National Children's Regional Medical Center Hangzhou China; ^2^ Department of Nephrology Children's Hospital, Zhejiang University School of Medicine, National Clinical Research Center for Child Health, National Children's Regional Medical Center Hangzhou China; ^3^ Center for Stem Cell and Regenerative Medicine, and Bone Marrow Transplantation Center of the First Affiliated Hospital Zhejiang University School of Medicine Hangzhou China; ^4^ Liangzhu Laboratory Zhejiang University Hangzhou China; ^5^ Department of Pathology Children's Hospital of Zhejiang University School of Medicine, National Clinical Research Center for Child Health Hangzhou China; ^6^ Zhejiang Provincial Key Lab for Tissue Engineering and Regenerative Medicine Dr. Li Dak Sum & Yip Yio Chin Center for Stem Cell and Regenerative Medicine Hangzhou China

Dear Editor,

Immunoglobulin A nephropathy (IgAN) and IgA vasculitis with nephritis (IgAVN) are closely related kidney diseases that share similar clinical and pathological characteristics.^1^ Single‐cell RNA sequencing (scRNA‐seq) of renal tissues from patients with IgAN and IgAVN identifies the similarities and distinctions between them, providing valuable insights into their pathogenesis, diagnosis, and treatments.

Kidney samples from pediatric patients with IgAN (*n* = 6) and IgAVN (*n* = 5), and from controls (*n* = 4) were collected for scRNA‐seq (Table S1 provides the detailed information of the 15 participants). Note that, 79 137 retained single cells were partitioned into 30 distinct clusters through uniform manifold approximation and projection (specific data details can be found in Tables [Link], [Link], [Link]). Fifteen major cell types in the kidney were identified (Figure 1A) relying on canonical markers associated with kidney cell populations (Figure 1B and Table S5). Initially, a comprehensive analysis of the average gene expression profiles was conducted for the IgAN and IgAVN groups in comparison to those in the control group (Figure 1C,D). Importantly, there was a significant overlap between the two conditions (IgAN vs. control and IgAVN vs. control), with 233 downregulated genes and 167 upregulated genes common to both IgAN and IgAVN (Figure 1E,F). The shared differentially expressed genes (DEGs) were analyzed using the Kyoto Encyclopedia of Genes and Genomes, which shed light on the shared molecular mechanisms in metabolic, secretory, and inflammatory pathways underlying IgAN and IgAVN (Figure 1G,H and Tables S6 and S7).

**FIGURE 1:**
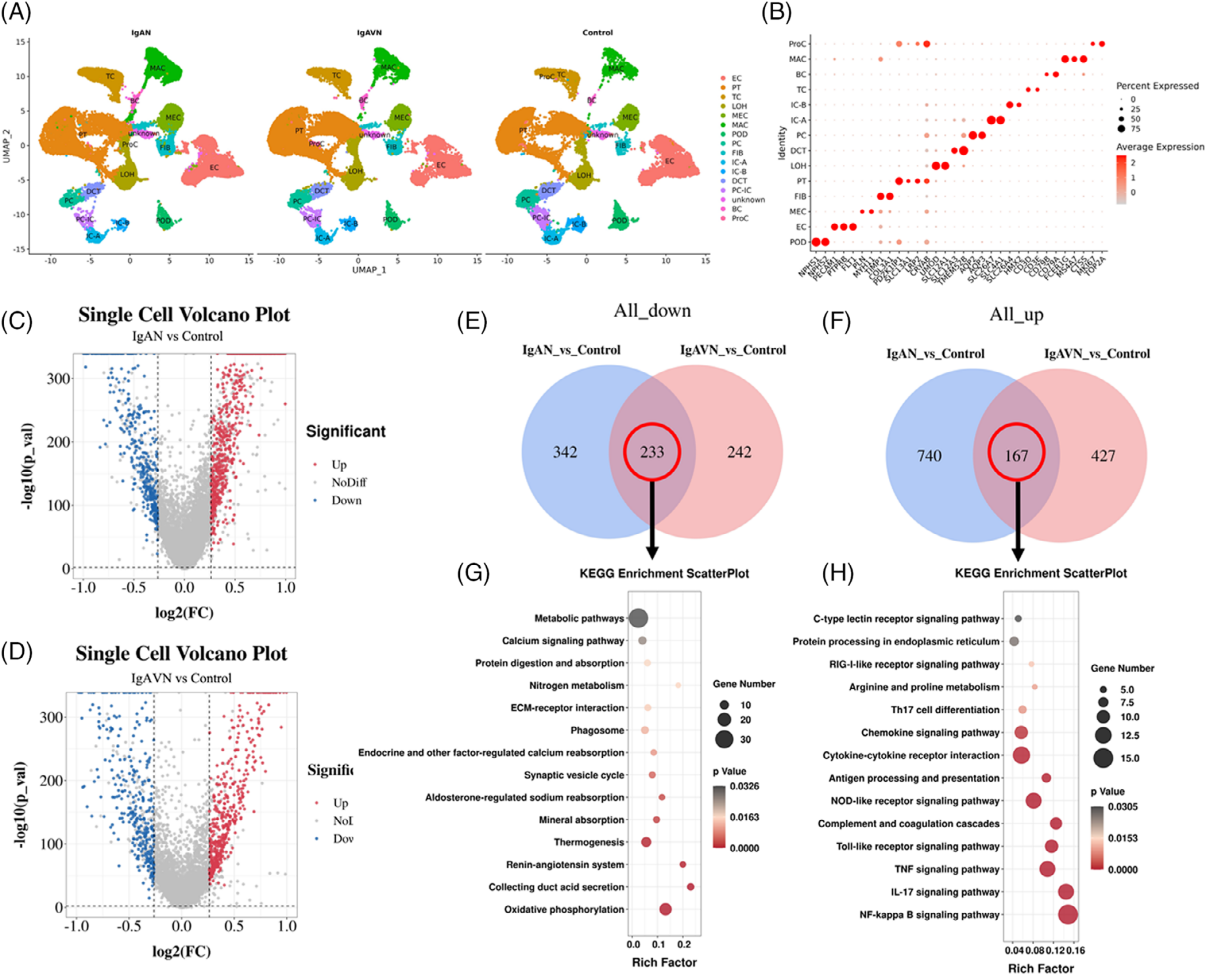
Visualization of the single‐cell RNA sequencing (scRNA‐seq) data and differences analysis at the holistic level. (A) The uniform manifold approximation and projection (UMAP) cluster map visually displays 15 distinct clusters representing major kidney cell types. Each dot on the map corresponds to an individual cell, and the colours represent specific cell types, with abbreviations such as POD (podocytes), EC (endothelial cells), MEC (mesangial cells), FIB (fibroblasts), PT (proximal tubular cells), LOH (loop of Henle), DCT (distal convoluted tubule), PC (principal cells), PC‐IC (transitional cells), IC‐A (intercalated cells type A), IC‐B (intercalated cells type B), BC (B cells), TC (T cells), MAC (macrophages) and ProC (proliferating cells). (B) The dot plot visualization illustrates the average expression of selected marker genes in each identified cell type and the percentage of cells within each type expressing these markers. (C, D) The volcano maps depict the results of differential gene analysis for immunoglobulin A nephropathy (IgAN) versus control and IgA vasculitis with nephritis (IgAVN) versus control. The blue dots indicate downregulated genes, the red dots indicate upregulated genes and the grey dots represent genes with no significant differences. (E, F) The Venn diagram illustrates the overlap of downregulated and upregulated differentially expressed genes (DEGs) in IgAN versus control and IgAVN versus control. (G, H) These scatterplots display the Kyoto Encyclopedia of Genes and Genomes (KEGG) pathway enrichment analysis results for downregulated and upregulated DEGs common to both IgAN and IgAVN.

Digging deeper into our investigation, we endeavoured to discern the nuanced distinctions between IgAN and IgAVN. Of particular interest was the analysis of cell type proportions, where we observed a conspicuous discrepancy in the prevalence of proximal tubule (PT) cells between the two diseases (Figure 2A). A notable reduction was observed in the proportion of PT cells in IgAN compared to in IgAVN (Figure 2B). Subgroup analysis of PT cells revealed a notably greater prevalence of injured PT cells and acute injured PT cells in IgAN groups than in IgAVN groups (Figure 2C,D). To uncover the underlying factors contributing to the significant disparities observed in PT cell proportions and susceptibility to injury between these two conditions, we analyzed the DEGs in PT cells between IgAN and IgAVN (Figure 2E,F). A list of DEGs for IgAN and IgAVN in PT cells is available in Table S8. Using Gene Ontology (GO) analysis, we found that the downregulated DEGs unique to IgAN in PT cells (Figure 2G, section “a”) were predominantly associated with energy metabolism processes (Figure 2I). Conversely, the downregulated DEGs specific to IgAVN in PT cells (Figure 2G, section “b”) were primarily linked to protein translation and growth regulation (Figure 3J). Furthermore, upregulated DEGs specific to IgAN (Figure 2H, section “c”) were primarily associated with processes such as cell adhesion, cell migration, cell‒cell junctions, angiogenesis and apoptotic processes (Figure 2K). In contrast, IgAVN displayed an increase in genes related to energy metabolism processes (Figure 2H, section “d”), particularly those associated with mitochondrial functions (Figure 2L). Table S9 (sheets 1−4) shows an extensive list of GO pathways and detailed statistical information within each group. These findings collectively point to a fundamental distinction in energy metabolism between IgAN and IgAVN.

**FIGURE 2:**
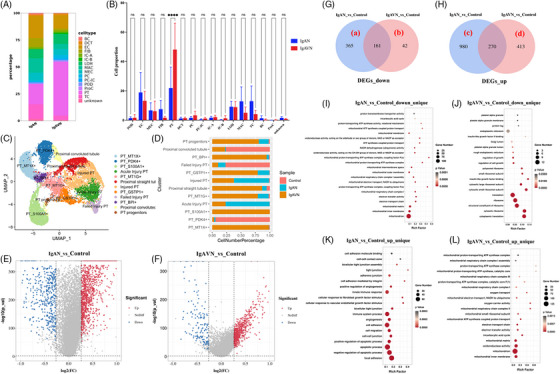
Comparative analysis of proximal tubule (PT) cells in immunoglobulin A nephropathy (IgAN) and IgA vasculitis with nephritis (IgAVN). (A) The composition of various cell types is depicted by distinct colours within their respective groups and samples. (B) A statistical evaluation of the cell type distribution in IgAN and IgAVN was conducted using the Mann‒Whitney U test, a nonparametric analysis, with statistical significance defined as *p* < .05. The statistical software GraphPad Prism 9.5.1 was used for this analysis. (C) The uniform manifold approximation and projection (UMAP) cluster map provides a visual representation of distinct cell clusters within PT cells. Each dot on the map represents an individual cell, with colours denoting specific cell subclasses. (D) The distribution of various cell subclasses is illustrated by color‐coded groups. (E, F) Volcano maps illustrating differential gene expression between the IgAN versus control and between the IgAVN versus control within PT cells. (G, H) Venn diagrams displaying downregulated and upregulated differentially expressed genes (DEGs) for IgAN versus control and IgAVN versus control in PT cells. (I&J) GO enrichment scatterplots highlighting the pathways enriched in downregulated DEGs unique to IgAN and downregulated DEGs unique to IgAVN. (K, L) GO enrichment scatterplots showing the pathways enriched in upregulated DEGs unique to IgAN and upregulated DEGs unique to IgAVN.

**FIGURE 3:**
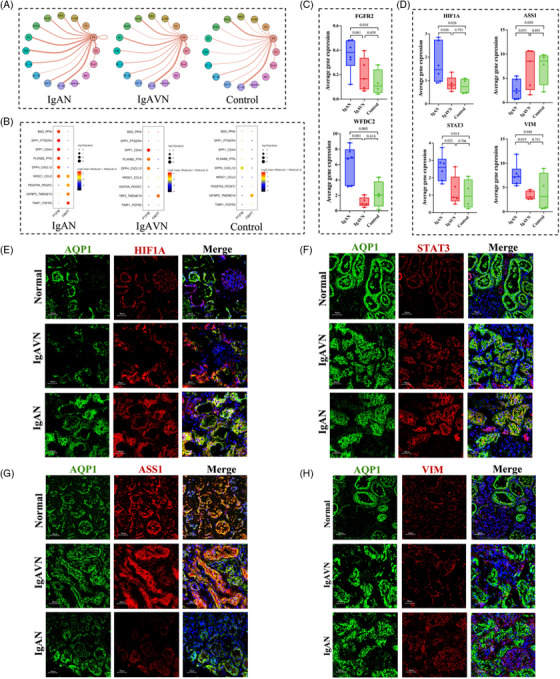
Cell communication analysis and differential gene expression in proximal tubule (PT) cells. (A) Network graphs illustrating interactions between PT cells and other cell types. Nodes represent specific cell types, and the thickness of connecting lines signifies the number of protein interactions between different cell types. (B) Dot plot diagrams displaying ligand‒receptor interactions in the immunoglobulin A nephropathy (IgAN), IgA vasculitis with nephritis (IgAVN), and control groups. (C) Average expression levels of fibroblast growth factor receptor 2 (FGFR2) and WAP four‐disulfide core domain 2 (WFDC2) in the IgAN, IgAVN and control groups. (D) Average gene expression levels of the HIF1A, STAT3, ASS1 and VIM genes in the IgAN, IgAVN, and control groups. (E–H) Immunofluorescence validation using human kidney tissue. AQP1 serves as a marker for proximal renal tubules (green, Proteintech, Cat No. 66805‐1‐Ig). Immunofluorescence staining for HIF1A (Proteintech, Cat No. 20960‐1‐AP), STAT3 (Proteintech, Cat No. 10253‐2‐AP), ASS1 (Proteintech, Cat No. 16210‐1‐AP) and VIM (Proteintech, Cat No. 10366‐1‐AP) proteins is shown in red. Magnification: 40×.

Subsequently, analysis of the CellPhoneDB revealed a notably heightened level of communication between PT cells and fibroblasts within the context of IgAN (Figure 3A), with specific ligand–receptor pairs implicated in these interactions (Figure 3B). One particularly noteworthy receptor involved in this dynamic interaction between fibroblasts and PT cells is fibroblast growth factor receptor 2, which is important in renal interstitial fibrosis by governing the transdifferentiation of tubular epithelial cells and interstitial fibroblasts (Figure 3C).^2^ Furthermore, we noted elevated expression of WAP four‐disulfide core domain 2 in IgAN patients (Figure 3C), which has recently emerged as a potential clinical prognostic biomarker for kidney disease and fibrosis.^3^, ^4^ Renal fibrosis is often synonymous with the gradual deterioration of renal function and the progression to end‐stage renal disease.^5^ Recently, Lv et al.^6^ reported that IgAN patients had higher pathological scores for complete tubular atrophy, interstitial fibrosis as well as interstitial and periglomerular inflammation compared to IgAVN patients, which is consistent with our data and strongly supports our conclusions.

To validate our results, we further analyzed genes related to energy metabolism and fibrosis in PT cells. Hypoxia‐inducible factor 1 subunit alpha (HIF1A) is a pivotal regulator of hypoxia adaptation, energy metabolism, apoptosis and epithelial–mesenchymal transition (EMT).^7^, ^8^ Indeed, our findings confirmed higher HIF1A expression in IgAN patients than in IgAVN patients and in the control group (Figure 3D). Notably, signal transducer and activator of transcription 3 (STAT3), an upstream activator of HIF1A associated with kidney repair,^9^, ^10^ exhibited a consistent expression pattern with that of HIF1A (Figure 3D). The intricate interplay between STAT3 and HIF1A orchestrates various processes, including cell metabolism, survival, programmed cell death, and fibrosis.^9^, ^10^ Moreover, we detected differential expression of known HIF1A target genes, including argininosuccinate synthase 1 (ASS1) and vimentin (VIM). ASS1, an enzyme in the urea cycle, detoxifies neurotoxic ammonia from protein breakdown and displayed heightened expression in IgAVN (Figure 3D). VIM, a marker of renal tubular EMT, exhibited increased expression in IgAN patients (Figure 3D). The validation of these protein expression levels through immunofluorescence analysis of human kidney tissues is presented in Figure 3E–H.

In conclusion, scRNA‐seq analysis of children's kidney samples has pinpointed notable disparities between IgAN and IgAVN, including defects in energy metabolism, variations in HIF1A gene expression, and heightened fibrosis levels in IgAN patients, offering more in‐depth data on the single‐cell transcriptome regarding the relationship between IgAN and IgAVN, potentially influencing future diagnostic approaches, treatment options, and predictive outcomes for patients with these conditions.

## AUTHOR CONTRIBUTIONS

Jianhua Mao and Guoji Guo contributed to the study's conception and design. Material preparation, data collection and analysis were performed by Qing Ye, Hanyan Meng, Fang Ye, Haidong Fu, Jingjing Wang, Fei Liu, Huijun Shen, Yu Bao Chunyue Feng, Xiaojing Zhang, Guoping Huang, Yi Xie and Manli Zhao. The first draft of the manuscript was written by Qing Ye and Hanyan Meng. Jianhua Mao and Guoji Guo contributed to discussing and interpreting the results and reviewed the final manuscript. All the authors commented on previous versions of the manuscript and read and approved the final manuscript.

## CONFLICT OF INTEREST STATEMENT

The authors declare no conflict of interest.

## FUNDING INFORMATION

This work was financially supported by the National Key R&D Program of China (2021YFC2702002), the National Natural Science Foundation of China (U20A20351; 82270741; 82000676), the Natural Science Foundation of Zhejiang Province (LY22H050001), the Key Project of Provincial Ministry Construction, Health Science and Technology Project Plan of Zhejiang Province (WKJ‐ZJ‐2128), the Key Research and Development Plan of Zhejiang Province (2021C03079), the Zhejiang Xinmiao Talents Program (2023R401208) and A Project Supported by Scientific Research Fund of Zhejiang University (XY2023011).

## ETHICS STATEMENT

The Ethics Committee of the Children's Hospital, Zhejiang University School of Medicine (2021‐IRB‐228), approved this study.

## PATIENT CONSENT STATEMENT

Informed consent was obtained from all patients involved in the study.

## Supporting information

Supporting Information

Supporting Information

Supporting Information

Supporting Information

Supporting Information

Supporting Information

Supporting Information

Supporting Information

Supporting Information

## Data Availability

The data are available upon reasonable request. All the data are presented in the manuscript. The raw experimental data will be made available upon reasonable request.
